# Narratives and mental health in the Covid-19 Pandemic

**DOI:** 10.1007/s44192-022-00013-2

**Published:** 2022-04-19

**Authors:** Marcelo Simões Mendes

**Affiliations:** Anhanguera University of Sao Paulo/Brazil, Rua Pastor Santino Soares Da Silva, 252. CEP, Sumaré, SP 13180-151 Brazil

**Keywords:** Narratives, Belief system, Psychic suffering

## Abstract

The rise in the flow of narratives is directly associated with technological advances in communication. In pandemic times, the narratives have produced profound consequences in many dimensions of human life, such as individuals' belief systems. Narratives operate as a background of the self's functioning and present substantial importance to personality development. On the other side, narratives can influence the functioning of the self's disorders. It occurs when narratives do not respect a hierarchical belief system in the individuals. The disrespect to the central beliefs that operate in the individuals' personalities often fosters a psychosomatic process that distances them from authentic contact with themselves. Consequently, it enhances mental suffering and psychic illness. The current paper aimed to discuss some intersections between narratives and states of psychic suffering.

## Introduction

The Covid-19 pandemic has specific characteristics which differ from other global catastrophes, such as the speed of information transmission [1] aligning with the construction of narratives propelled. The narratives have interfered in various domains of health [2, 3], social life [4–7], and mental life [8]. Besides, the intense flow of narratives during the Covid-19 pandemic has confused people in many domains of treatments, vaccines, and measures to contain the spread of the virus.

Although, narratives have other vital functions than distorting reality and fostering fear in the population with false or partial information. Narratives are aligned with human development in different ways. Even fictional (imaginary world) or nonfictional (real-life story), the narratives comprehend an ontological semantic core [9]. Thus, the narratives operate as a background of the self's functioning and support rewriting it [10]. Therefore, the narratives significantly influence the development of personality, healthy or disturbing functioning. The current paper aimed to discuss some intersections between narratives and states of psychic suffering. In addition, it proposed to reflect how a global state of suffering and the advances in information technology can influence destructive or healthy attitudes.

## Narratives, technology, and meanings in pandemic times

Despite the advances in COVID-19’s vaccines program worldwide, Kalin [11] has warned of devasting effects of the pandemic on mental health for years. The author presented some specific stress-related disorders associated with the SARS-CoV-2 pandemic: fear to get sick and die, worries to infect other people, social isolation, loss of job, and income [11]. Especially in moments of suffering, words and narratives have their impact enhanced. Besides, loneliness and anxiety were associated with increased suicidal ideation during the novel coronavirus pandemic [12]. It is crucial to understand the relationships between mental disorders, the core meanings of words, and narratives. Meaning-making processes and self-interpretation are aligned with current dynamic and transdiagnostic approaches to treating psychopathological symptoms, including psychosis [13].

The study of Gubrium and Gubrium [14] brought to light vital content in public debate during pandemic times. In times when technology improves the aesthetics of communication, authors warned of the need to understand the meaning of words and how they have been used in speeches or assemblages of narratives [14]. This process is guided by the paradigm of complexity [15], in which distinct parts must be carefully considered in the complete analysis.

The connectivity, speed of information, and broad access for content production are specific and significant characteristics to consider in the Covid-19 pandemic crisis. The possibility to confirm an affirmation or negation is not always constructive and beneficial. The attribution of meaning in the belief system of individuals does not work in the same way and with the same speed as the information is produced. Besides, dealing with many possibilities of meanings or truth beyond the narratives is often complicated and exhausting. Psychological preparation is required because the flow of meanings can contribute to the best attitudes but also influence the worst.

## Narratives, beliefs, and mental health

Among the most elementary foundations of the human psyche, belief systems present essential contributions to understanding individuals’ mental functioning and personality development. From the trajectory of people’s lives, it is possible to notice that they develop and qualify their beliefs from various aspects of their experiences. Beliefs do not have the same meaning and value [16] for everyone. Some core beliefs do not have the same value as peripheral beliefs [16]. From the distinction of each person's values, it is rational to infer a belief system functioning by a hierarchical structure in which some fundamental beliefs support others.

A priori, the hierarchical belief system cannot be understood as a positive or negative attitude because it describes a structural functioning. Positive or negative attitudes are consequences of a complex functioning in which beliefs comprehend a structural part of attitude's phenomenon. The differentiation of meanings and values in the belief’ system is not an exceptional condition for some individuals. Indeed, it is a fundamental and elementary principle for developing the individuals' singularities from a specific to a broad perspective.

Merit is one of the central beliefs of a hierarchical system, and its development influences crucial domains of personality and mental health, such as self-esteem, identity, and empathy. An individual can corrupt or mischaracterize his personal merit beliefs by influencing narratives. In a hypothetical case, an individual who transfers all his responsibilities to other agents, institutions, or conditions removes valuable resources to improve some self's characteristics. The narratives that totalized the social inequalities in the collective domains offer an 'ideal' rhetorical condition for someone hiding their intimacy in the social dimension. Besides, the generalization of social inequalities as an end in themselves inserts individualities as a simplistic attribute in the real modifications of life conditions.

In addition, the distinction of meaning and values in the belief' system permits individuals to recognize the elements of reality more properly and less propitious to alienated positions. The production of opposite or contradicting narratives potentiates uncertainties, leaving individuals more conducive to driving that somehow calms their fears. It is a propitious scenario for the implementation of authoritarianism. In the current Covid-19 pandemic, when Big Techs still have hard influences on the production and development of narratives, alienated' beliefs and positions reveal a dangerous condition to mental control and mass psychosis [17]. The control of publicity is one of the most effective strategies to implement totalitarianism [18].

Narratives can corrupt the beliefs system of individuals and further enhance inequalities when the conditions of real impediments are not differentiated from supposed or ideological impediments. Thus, it is crucial to understand how the narratives impact individuals' belief systems and vice versa. On the one side, narratives can contribute to the healthy development of individuals' personalities. However, disrespecting the central beliefs that operate in their personalities fosters a psychosomatic process that distances them from authentic contact with themselves. Consequently, it enhances mental suffering and psychic illness.

## Final considerations

It is understood that the clarity and appropriation of the influences of narratives in mental functioning can contribute to better comprehension and diagnostics on many fronts associated with mental suffering and social intolerance. The denial of a hierarchical belief system of individuals exposes them to conditions of psychic suffering. Furthermore, it fosters more inequalities and social tensions because it distorts the individuals' singularities so that there is no possibility of healthy social participation. Besides, narratives can foster the sensation of uncertainty and influence people to become more conducive to accepting everything, even destructive rules.

Narratives significantly influence the self’s development and the relationships between individuals worldwide. They can even influence the functioning of the self positively and healthily. However, understanding how they affect the individual and the collective in which they participate is crucial, especially as mental suffering in the pandemic has increased exponentially.

## Data Availability

Not applicable.
